# NS-11021 Modulates Cancer-Associated Processes Independently of BK Channels in Melanoma and Pancreatic Duct Adenocarcinoma Cell Lines

**DOI:** 10.3390/cancers13236144

**Published:** 2021-12-06

**Authors:** Alessia Remigante, Paolo Zuccolini, Raffaella Barbieri, Loretta Ferrera, Rossana Morabito, Paola Gavazzo, Michael Pusch, Cristiana Picco

**Affiliations:** 1 Biophysics Institute, National Research Council, 16149 Genoa, Italy; alessia.remigante@ibf.cnr.it (A.R.); paolo.zuccolini@ibf.cnr.it (P.Z.); raffaella.barbieri@ibf.cnr.it (R.B.); paola.gavazzo@ibf.cnr.it (P.G.); cristiana.picco@ibf.cnr.it (C.P.); 2 Department of Chemical, Biological, Pharmaceutical and Environmental Sciences, University of Messina, 98166 Messina, Italy; rmorabito@unime.it; 3 U.O.C. Genetica Medica, Istituto di Ricovero e Cura a Carattere Scientifico (IRCCS), Istituto Giannina Gaslini, 16147 Genoa, Italy; loretta.ferrera@unige.it

**Keywords:** BK channel, BK openers, calcium, IGR39 cells, Panc-1 cells, cancers

## Abstract

**Simple Summary:**

Potassium channels permit the selective passage of K^+^ ions across the cell me brane and are important for setting the membrane potential and for the transmission of electrical signals in all cells. Ca^2+^ activated K^+^ channels provide a means to couple intracellular calcium signaling to changes of the membrane potential. Among these, the large-conductance Ca^2+^-activated K^+^ channel, known as BK, has been proposed to be involved in several cancer-associated processes. In the present work, we tested various BK channel activators for anti-cancer effects in melanoma and pancreatic duct carcinoma cell lines. Only one of the activators (NS-11021) had effects on cancer-associated processes. However, the compound, as a side-effect, also increased the intracellular Ca^2+^ concentration independently of BK channel activation. Overall, we conclude that the activation of the BK channel by itself is not sufficient to produce beneficial anti-cancer effects.

**Abstract:**

Potassium channels have emerged as regulators of carcinogenesis, thus introducing possible new therapeutic strategies in the fight against cancer. In particular, the large-conductance Ca^2+^-activated K^+^ channel, often referred to as BK channel, is involved in several cancer-associated processes. Here, we investigated the effects of different BK activators, NS-11021, NS-19504, and BMS-191011, in IGR39 (primary melanoma cell line) and Panc-1 (primary pancreatic duct carcinoma cell line), highly expressing the channel, and in IGR37 (metastatic melanoma cell line) that barely express BK. Our data showed that NS-11021 and NS-19504 potently activated BK channels in IGR39 and Panc-1 cells, while no effect on channel activation was detected in IGR37 cells. On the contrary, BK channel activator BMS-191011 was less effective. However, only NS-11021 showed significant effects in cancer-associated processes, such as cell survival, migration, and proliferation in these cancer cell lines. Moreover, NS-11021 led to an increase of intracellular Ca^2+^ concentration, independent of BK channel activation, thus complicating any interpretation of its role in the regulation of cancer-associated mechanisms. Overall, we conclude that the activation of the BK channel by itself is not sufficient to produce beneficial anti-cancer effects in the melanoma and PDAC cell lines examined. Importantly, our results raise an alarm flag regarding the use of presumably specific BK channel openers as anti-cancer agents.

## 1. Introduction

The Big Potassium (BK) voltage- and Ca^2+^ activated ion channel is expressed in a wide variety of cells and is commonly known by different names (Maxi-K, Slo-1, KCa1.1) [1,2]. BK channels are mostly localized in the plasma membrane, although they have been found also in the inner mitochondrial membrane [3]. Each BK subunit of the homotetrameric assembly is formed by seven transmembrane segments (S0-S6), containing a voltage sensor domain (VSD, S1-S4), a pore-gate domain (PGD, S5-S6), and a cytosolic tail domain (CTD) that harbors the Ca^2+^ binding sites [4,5]. BK channels modulate numerous physiological processes in excitable and non-excitable tissues and take part in forming molecular signaling complexes [6]. Several associated subunits co-assemble with the main pore-forming complex in a variable manner dependent on cell type [4]. Because of the large number of protein interactions and activating factors influencing BK channel function, including intracellular Ca^2+^, membrane voltage, pH, and phosphorylation [7], as well as G-proteins, steroid hormones, and various drugs [8,9], it is generally difficult to predict their effective role in different cell types [10].

BK channels allow potassium ions to pass selectively, and their gating is subject to physio-pathological and pharmacological regulation [8]. In this regard, the BK channel has been a target of cancer therapeutic drug discovery, although its role is complex and may not be universal. BK channels are over-expressed in some cancers [11]. It is generally hypothesized that a large BK conductance favors the maintenance of a strongly polarized (i.e., negative) membrane potential (Em). Many pieces of evidence have demonstrated a large variability of Em in cancer cells [12]. In fact, alterations in Em (depolarization or hyperpolarization) have been proposed to play a crucial role in controlling the cell cycle, thus suggesting a relevance of BK channel in cancer proliferation, invasion, and metastasis [12]. In this regard, in some studies, activation of BK channels has been reported to slow proliferation and invasion of breast cancer cells, or in glioblastoma cells [11,13,14,15,16]. In contrast, in other studies, anti-cancer action has been described for inhibition of BK channels, namely, in metastatic breast cancer cells, as well as in endometrial cancer cells [17,18,19]. In light of the involvement of BK channel in various pathophysiological conditions, the biophysical mechanisms underlying its regulation have been extensively studied, and BK openers are sought as novel avenues to the treatment of diseases, including stroke, epilepsy, asthma, arterial ischemic heart disease, and cancer. These activators include NS-11021, NS-19504, and BMS-191011 [13,20,21,22,23].

Pancreatic adenocarcinoma and melanoma are two of the deadliest forms of cancer [24,25], and unfortunately many molecular details addressing their malignant transformation are still missing. Therefore, a better characterization of the key drivers and pathways involved is urgent, in order to find prognostic molecular markers and design new therapeutic opportunities finalized to reduce metastasis occurrence. The effect of BK channel activators in both melanoma and pancreatic duct adenocarcinoma has not been evaluated thus far, and indeed the present investigation aims to clarify the Yin and Yang role of BK channel in these types of cancer.

**Figure 1:**
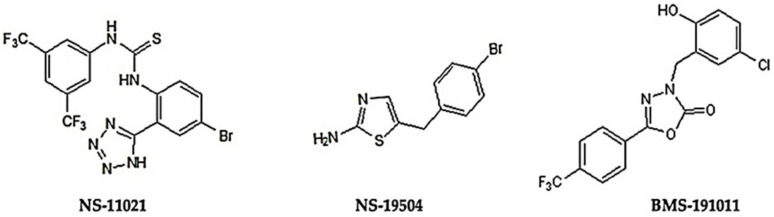
Chemical structures of tested BK channel openers.

Since it is widely assumed that BK channels could modulate cancer progression, in the present study, the effects related to BK channel activation in two different cancer cell lines (melanoma and pancreatic duct carcinoma) were investigated. First, we tested whether the chosen activators (Figure 1) could directly modulate BK channel activity. Second, we explored whether BK channel openers could affect any of the cancer-specific mechanisms, and thereby reduce cell viability, migration, or proliferation.

## 2. Materials and Methods

### 2.1. Cell Culture

The primary and metastatic melanoma cell lines, respectively, IGR39 and IGR37, obtained from the same patient (Deutsche Sammlung von Mikroorganismen und Zellkulturen GmbH, Braunschweig, Germany), were cultured in DMEM high-glucose medium (Euroclone, Pero, Italy) supplemented with 10% fetal bovine serum (Euroclone), 2 mM L-glutamine, 100 U/mL penicillin, 100 µg/mL streptomycin, 1% vitamin mix, and 1% non-essential amino acids. Panc-1 cells, primary pancreatic duct carcinoma cells (PDCA), obtained from American Type Cell Culture Collection (ATCC, Manassas, VA, United States) were cultured in DMEM high-glucose (Euroclone) supplemented with 10% fetal bovine serum (Euroclone), 4 mM L-glutamine, 100 U/mL penicillin, and 100 µg/mL streptomycin. All cells were cultured at 37 °C, 5% CO_2_, 95% air atmosphere, and 100% humidity. Subcultures were routinely established every second to third day by seeding the cells into T-25 (cm^2^) culture flasks following trypsin/ethylenediaminetetraacetic acid (EDTA) treatment.

### 2.2. Chemicals

BK channel openers were purchased from Tocris Bioscience (Bristol, UK), whereas paxilline (PAX) was purchased from Merck (Milan, Italy). All substances, prepared in dimethyl sulfoxide (DMSO), were diluted from a 50 mM stock solution and stored at −20 °C. DMSO never exceeded 0.1% in the final experimental solutions.

### 2.3. Patch Clamp Recording

Cells were transferred to Petri dishes (3.5 cm diameter), and electrophysiology measurements were performed 24–36 h later. Selected cells were tested using the whole cell patch clamp technique as previously described [26]. The resistance of the glass pipettes was 3–5 MOhm when filled with the intracellular pipette solution containing (in mM): 8 NaCl, 100 K-aspartate, 40 KCl, 2 CaCl_2_, 10 EGTA, 10 HEPES (pH 7.3, adjusted with KOH and 300 mOsm/kg H_2_O; free Ca^2+^ concentration ≈20 nM). In Table 1, the extracellular solutions used for the recordings are reported. The standard current-voltage protocol for stimulation consisted of 500 ms long voltage steps from −80 to +120 mV in 20 mV increments, starting from a holding potential of −50 mV. Cell response to the various stimuli was monitored using the “time course protocol”, which consisted of a “staircase” of 50 ms pulses to −100, −50, 0, +50, and +100 mV, administrated every 5 s.

The time-course protocol allowed also for the monitoring of series resistance and membrane capacitance by analyzing the initial step response of the pulse to −100 mV. Currents were corrected offline for series resistance errors. Membrane currents were filtered at 10 kHz and digitized at 50 kHz with a National Instruments DAQ interface or an ITC16 interface (InstruTech, Longmont, CO, USA), employing the GePulse acquisition program (freely available at http://users.ge.ibf.cnr.it/pusch/programs-mik.htm, accessed on 1 January 2019). For data analysis, the Ana analysis program (http://users.ge.ibf.cnr.it/pusch/programs-mik.htm, accessed on 1 January 2019 was used. The current values (pA) were normalized to the membrane capacitance (pF) to obtain the current density (pA/pF). Each dataset was obtained from cells from at least three independent subcultures with control experiments in cells of the same subculture.

### 2.4. Ca^2+^ Imaging with FURA-2-AM

Measurements of intracellular Ca^2+^ ([Ca^2+^]_i_) were performed using the fluorescent Ca^2+^ indicator FURA-2-AM. Cells were loaded with 5 μM FURA-2-AM dissolved in extracellular solution containing 0.1% of pluronic acid to improve dye uptake, for 45 min at 37 °C. Cell coverslip was placed on the stage of an inverted Nikon TE200 fluorescence microscope (Nikon, Tokyo, Japan) equipped with a dual excitation fluorometric Ca^2+^ imaging system (Hamamatsu, Sunayama-Cho, Japan). Cells were excited at 340 and 380 nm at a sampling rate of 0.5 Hz, and fluorescence emission, measured at 510 nm, was acquired with a digital CCD camera (Hamamatsu C4742-95-12ER). The external solutions were the same used in patch-clamp experiments. The fluorescence ratio F340/F380 was used to estimate [Ca^2+^]_i_ changes. Monochromator settings, chopper frequency, and data acquisition were controlled by a specific software (Aquacosmos/Ratio U7501-01, Hamamatsu). [Ca^2+^]_i_ was calculated according to Grynkiewicz et al. [27], using a K_D_ of 140 nM for the Ca^2+^/FURA-2 complex. Since a generic ionic indicator produces an appreciable response in a range of concentrations between 0.1× Kd and 10× Kd, the dynamic range of the intracellular calcium variations can be estimated between 20 nM and 1.5 µM. Each dataset was obtained from cells from at least three independent subcultures.

### 2.5. Cell Viability Assay

Cells were seeded into 96-well plates (3 × 10^3^ cells per well), and the day after were exposed to 20 μM NS-11021, or alternatively 20 μM NS-19504 for 72 h. After incubation, 20 μL of 3-(4,5-dimethylthiazol-2-yl)-2,5-diphenyltetrazolium bromide solution (MTT, Promega, Milano, Italy) was added to each well and incubated for 2 h at 37 °C, 5% CO_2_. Cell viability was calculated from the ratio of absorbance at 570 nm in the drug-treated versus control cells [28]. Each dataset was obtained from cells from at least three independent subcultures; control experiments were from cells of the same subculture. 

### 2.6. Wound Healing Assay

Cells were seeded in Petri dishes (3.5 cm diameter) and maintained overnight. After cells reached confluence, a sterile 200 μL pipette tip was used to scratch the cells vertically in order to get a cell-free straight line. PBS was used to rinse off the castoff cells. Then, cells were incubated with various concentrations of NS-11021 (20 μM) or NS-19504 (20 μM) for 24 h, and simultaneously they were exposed to 10 μM cytosine arabinoside (ARAc) to inhibit cell proliferation [29]. Each dataset was obtained from cells from at least three independent subcultures; control experiments were performed in cells from the same subculture.

### 2.7. Cell Proliferation Assay

Cell proliferation was assessed by xCELLigence RTCA MP System (Roche, Monza, Italy) that monitors cellular events in real time by measuring electrical impedance across interdigitated gold micro-electrodes integrated on the bottom of tissue culture plates. The impedance measurement provides quantitative information about the biological status of the cells, including cell number. Cell-sensor impedance is expressed as an arbitrary unit called Cell Index. In order to determine the Cell Index, we seeded cells into 100 μL of standard medium in 96× microtiter plates (E-Plate-Roche, Roche, Monza, Italy). Background impedance was determined using 50 μL of standard medium. Cell attachment, spreading, and proliferation were monitored every 15 min using the xCELLigence system. After 24 h, 20 μM NS-11021 and 20 μM NS-19504 were added, and the mixture was incubated for 72 h. Experimental results were elaborated using RTCASoftware 1.2 that calculate the population doubling by fitting the curve to an exponential equation. The number of cells seeded was the same for every cell type (IGR37, IGR39 and Panc-1).

### 2.8. Statistics

All data are expressed as arithmetic means ± standard errors of the mean. For statistical analysis and graphics, GraphPad Prism (version 8.0, GraphPad Software, San Diego, CA, USA) and Excel (Version 2019, Microsoft, Redmond, WA, USA) software were used. Significant differences between mean values were determined by the unpaired Student’s *t*-test or ANOVA Dunnet’s post-test, as appropriate. Statistically significant differences were determined at * *p* < 0.05, ** *p* < 0.01, *** *p* < 0.001; (*n*) corresponds to the number of independent measurements.

## 3. Results

### 3.1. BK Channel Activators: NS-11021 and NS-19504

**Figure 2:**
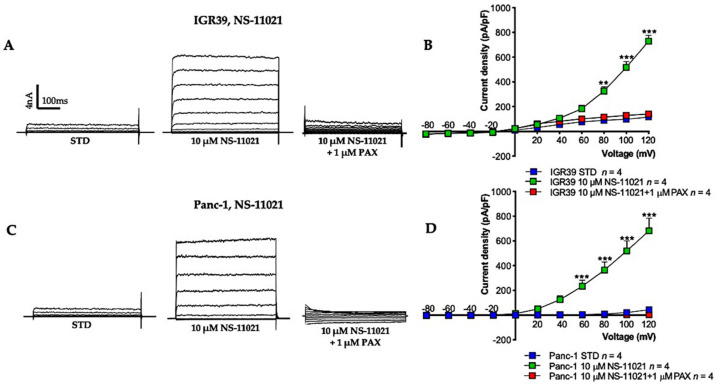
Effect of NS-11021 on membrane current in IGR39 and Panc-1 cells. Representative current traces obtained before and after NS-11021 addition, and after additional application of 1 µM paxilline in cells stimulated with voltage increments of +20 mV from −80 to +120 mV applied from a holding potential of -50 mV in (**A**) IGR39 and in (**C**) Panc-1. The duration of the voltage steps was 500 ms. Current density-voltage relationship measured in whole-cell patch-clamp experiments (**B**) in IGR39 and (**D**) in Panc-1. ** *p* < 0.01 and *** *p* < 0.001 versus STD condition, as determined by paired Student’s *t*-test. (n) refers to the number of cells.

Previous studies have demonstrated that BK channels are abundantly expressed in both IGR39 [30] and Panc-1 cells [31]. In order to investigate how specific BK channel activators can influence the biological properties of these two different cancer cell lines, we studied their biophysical properties using the patch-clamp technique in the whole-cell configuration. Perfusion of 10 µM NS-11021 elicited very large outward currents in IGR39 cells (Figure 2A,B), with a 26-fold increase at +100 mV (*p* < 0.01 and *p* < 0.001, *n* = 4) with respect to the control condition. Likewise, NS-11021 increased outward currents at membrane potentials more positive than +20 mV in Panc-1 cells (Figure 2C,D). At +100 mV, the outward current was increased approximately 15-fold (*p* < 0.01 and *p* < 0.001, *n* = 4). In both cell lines, currents slowly recovered almost to the initial level after washout of NS-11021. These results suggested the activated currents are predominantly carried by BK channels. To confirm this hypothesis, we employed a specific BK channel inhibitor, i.e., paxilline (PAX) [32]. As observed, 1 µM PAX reduced the BK channel openers-activated current by about 80% (Figure 2B,D). 

**Figure 3:**
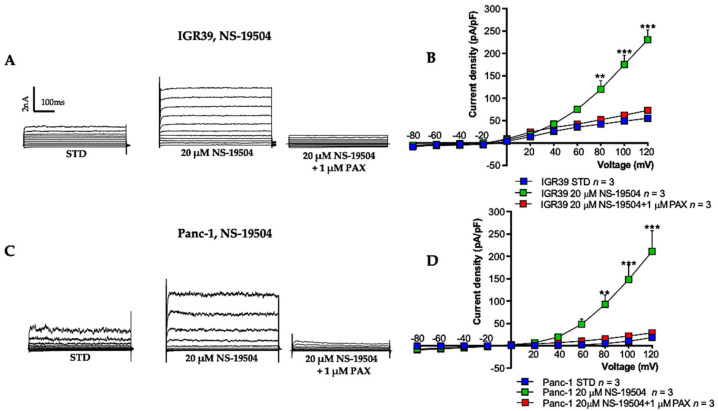
Effect of NS-19504 on membrane current in IGR39 and Panc-1 cells. Representative current traces obtained before and after NS-19504 addition, and after additional application of 1 µM paxilline in cells stimulated with voltage increments of +20 mV from −80 to +120 mV applied from a holding potential of −50 mV in (**A**) IGR39 and in (**C**) Panc-1. The duration of the voltage steps was 500 ms. Current density-to-voltage relationship measured in whole-cell patch-clamp experiments (**B**) in IGR39 and (**D**) in Panc-1. ** *p* < 0.01 and *** *p* < 0.001 versus STD condition, as determined by paired Student’s *t*-test. (n) refers to the number of cells.

Similarly, application of 20 µM NS-19504 induced an increase of the outward currents in both cell lines. Specifically, the current density at +100 mV was increased approximately fivefold (*p* < 0.01 and *p* < 0.001, *n* = 3) in IGR39 cells (Figure 3A,B), and sevenfold (*p* < 0.01, *n* = 3) in Panc-1 cells (Figure 3C,D). The effect of NS-19504 is largely reversible, as washing the cells with STD solution restored the voltage-dependent current to the initial conditions (Figure S1). In addition, to determine whether NS-19504-induced increase in outward current was specifically due to activation of BK channels, we also applied the blocker paxilline (1 µM) (Figure 3B,D).

**Figure 4:**
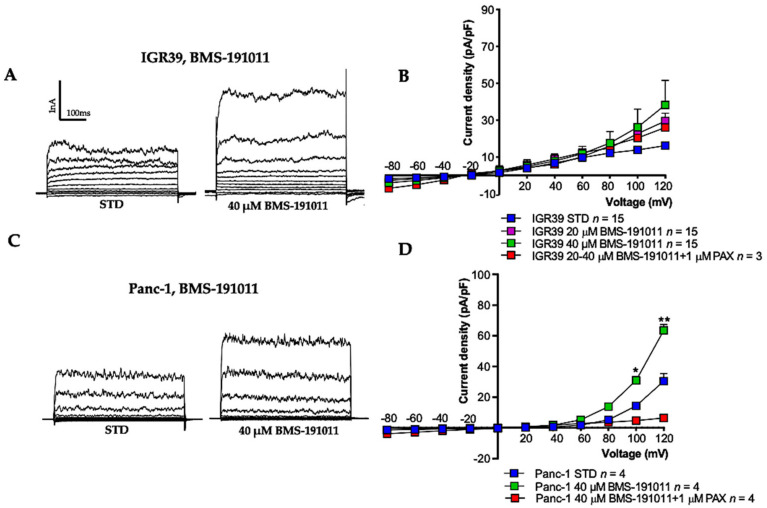
Effect of BMS-191011 on membrane current in IGR39 and Panc-1 cells. Representative current traces obtained before and after BMS-191011 addition in cells stimulated with voltage increments of +20 mV from −80 to +120 mV applied from a holding potential of −50 mV in (**A**) IGR39, or alternatively in (**C**) Panc-1. Current density–voltage relationship measured in whole-cell patch-clamp experiments (**B**) in IGR39 and (**D**) in Panc-1. Exposure to 40 µM BMS-191011 induced a moderate BK current in IGR39 cells and only a slight activation in Panc-1 cells. * *p* < 0.05, ** *p* < 0.01 versus STD condition, as determined by paired Student’s *t*-test. (n) refers to the number of cells. Note that in this figure, a different range of current density is indicated to that of Figure 2 and Figure 3.

Next, we tested another putatively specific BK channel opener, namely, BMS-191011 [13]. Surprisingly, BMS-191011 resulted in an increase of outward currents in less than 50% of all analyzed IGR39 cells (Figure 4A,B), with a modest degree of activation. In addition, in all cells showing activation of outward currents, BMS-191011 led to the parallel activation of inward currents at negative voltages, most probably mediated by KCa3.1 channels [30], possibly reflecting an increase of intracellular calcium levels. In Panc-1 cells, BMS-191011 resulted in a slight activation of outward currents (Figure 4C,D). Because of the limited activity of BMS-191011 on BK mediated currents, we did not employ the compound in further assays.

### 3.2. BK Channel Activation and Cell Viability in IGR39 and Panc-1 Cells

**Figure 5:**
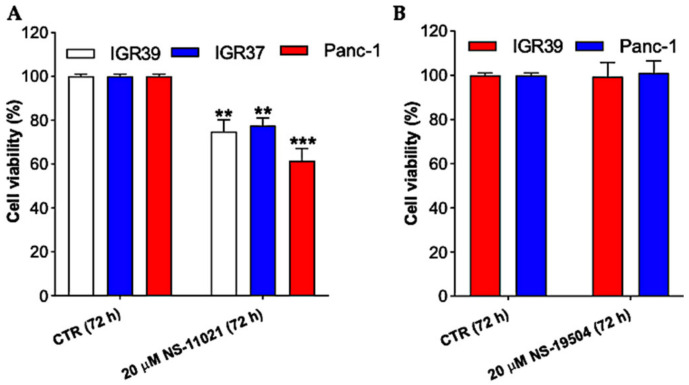
In vitro effects of BK channel openers on cell viability. Cells were treated with (**A**) 20 µM NS-11021 or (**B**) 20 µM NS-19504 for 24 h. The 0.1% DMSO (maximal percentage used in the treatments) had no obvious cytotoxicity. ** *p* < 0.01 and *** *p* < 0.001 versus control (CTR) as determined by one-way ANOVA followed by Dunnet’s post hoc test (*n* = 3).

To test whether the application of BK channel activators could induce cell death, we investigated the effects of NS-11021 and NS-19504 on cell viability in IGR39 and Panc-1 cells. Treatment with 20 µM NS-11021 for 72 h reduced viability of IGR39 and Panc-1 cells in comparison to their controls. In particular, NS-11021 exposure induced cell death that was statistically significant in comparison to the untreated group (*p* < 0.01 and *p* < 0.001, *n* = 3) in both cell lines (Figure 5A). To ensure that NS-11021-induced cell death was specifically caused by opening of BK channels, we used a metastatic melanoma cell line, in which BK channels are expressed 1400-fold less compared to their primary counterpart IGR39 [30]. Surprisingly, NS-11021 treatment induced cell death in IGR37 cells to a very similar extent (Figure 5A). These findings suggest the effect of NS-11021 is not specifically dependent on the opening of BK channels. Conversely, NS-19504 did not show any effect in cell viability assay (Figure 5B).

### 3.3. Effect of BK Channel Activators on Migration in IGR39 and Panc-1 Cells

**Figure 6:**
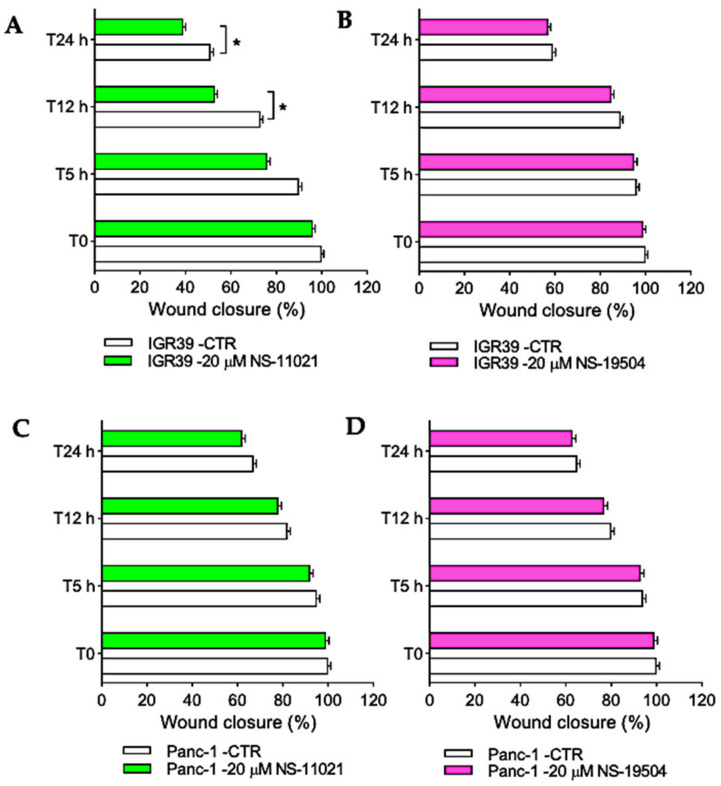
In vitro effects of BK channel openers on cell migration. IGR39 and Panc-1 cells were incubated with 20 µM NS-11021 (**A**,**C**) or 20 µM NS-19504 (**B**,**D**) for 24 h. The 0.1% DMSO (maximal percentage used in the treatments) was not significantly different versus control; * *p* < 0.05 versus control as determined by one-way ANOVA followed by Dunnet’s post hoc test (*n* = 3).

The majority of oncologic patients die for the consequences of cancer metastasis, and one critical step of metastasis is migration. In this regard, we verified whether the BK channel activators could affect migration in both IGR39 and Panc-1 cells. In these experiments, the antimitotic agent ARAc was employed to eliminate confounding effects of proliferation [29]. The exposure of NS-11021 prevented or slowed migration of IGR39 cells within 12 and 24 h (Figure 6A). Conversely, no effect was detected in Panc-1 cells (Figure 6C). Similarly, the treatment with NS-19504 activator did not affect cell migration in both analyzed cell lines (Figure 6B,D).

### 3.4. BK Channel Openers and Cell Proliferation in IGR39 and Panc-1 Cells

**Figure 7:**
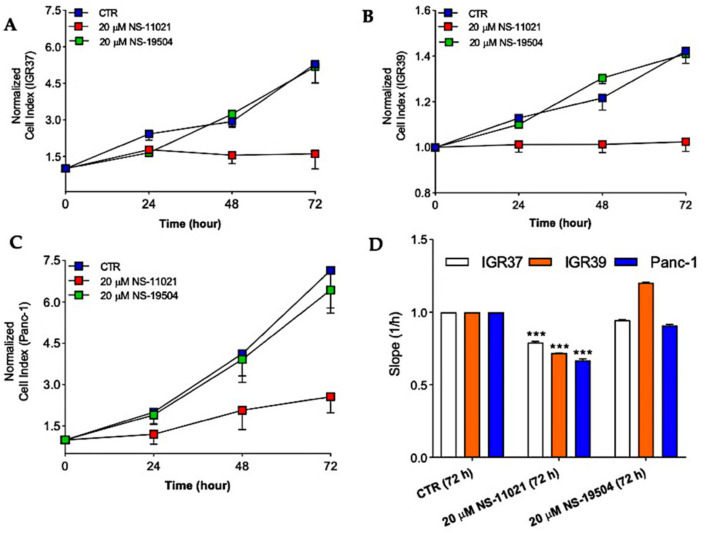
Real-time cell proliferation, monitored by the xCELLigence RTCA DP Instrument. Cell index (CI) curves of cellular proliferation of (**A**) IGR37, (**B**) IGR39, and (**C**) Panc-1 obtained after treatment for 72 h with 20 µM NS-11021 or NS-19504. (**D**) Cell proliferation has also been quantified by the slope of curves (CI) calculated by RTCA 1.2 software. The 0.1% DMSO (maximal percentage used in the treatments) had no obvious cytotoxicity. *** *p* < 0.001 versus control as determined by one-way ANOVA followed by Dunnet’s post hoc test (*n* = 3).

Uncontrolled proliferation is another of the main features of cancer cells. Thus, we next tested whether the BK channel activators could affect cell proliferation in IGR39 and Panc-1 cells. Our data (Figure 7) indicate that the treatment with NS-11021 for 72 h reduces the proliferation in both IGR39 and Panc-1, but also in IGR37 cells (negative control). Thus, the effect is unlikely mediated by BK channel opening. Conversely, NS-19504 did not affect proliferation in either cell line.

### 3.5. Measurement of Intracellular Ca^2+^ Levels in IGR39 and Panc-1 Cells

**Figure 8:**
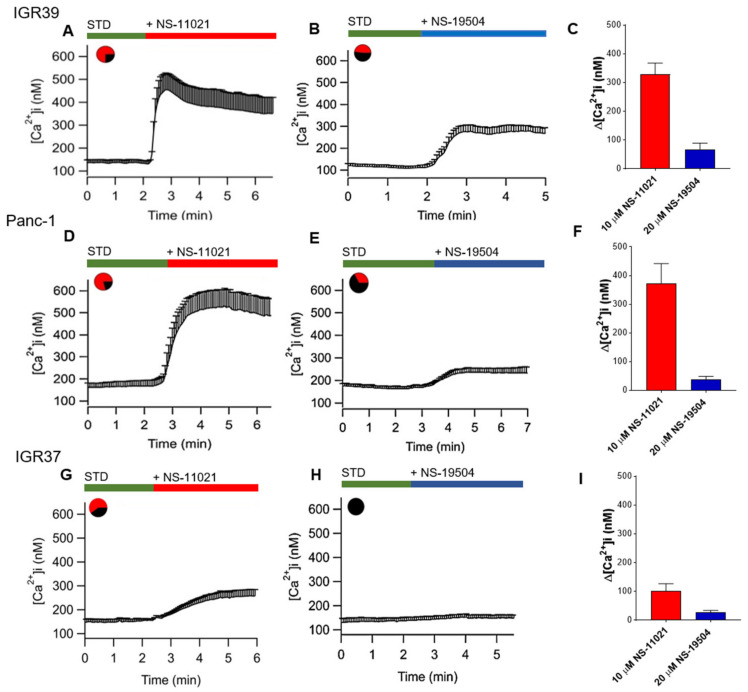
Effects of NS-11021 and NS-19504 on intracellular calcium. Average [Ca^2+^]_i_ signal in IGR39 and Panc-1 cells after the application of 10 µM NS-11021 or 20 µM NS-19054. Horizontal bars indicate the time period of exposure. Average traces were obtained from *n* = 53 cells ((**A**), IGR39), *n* = 57 cells ((**D**), Panc-1), and *n* = 33 cells ((**G**), IGR37) for NS-11021; *n* = 49 cells ((**B**), IGR39), *n* = 52 cells ((**E**), Panc-1), *n* = 32 cells ((**H**), IGR37) for NS-19504 in at least 5 independent experiments. Pie charts indicate the percentage of responsive cells (75% in IGR39, 79% in Panc-1, 54% in IGR37 for NS-11021, and 48% in IGR39, 33% in Panc-1 for NS-19054). Histogram (**C**,**F**,**I**) of mean data ± SE for both cell lines. IGR39: 328 ± 72 nM, 168 ± 33 nM; Panc-1: 372 ± 80 nM, 75 ± 21 nM; IGR37: 110 ± 22 nM, 14 ± 12 nM for NS-11021 and NS-19504.

**Figure 9:**
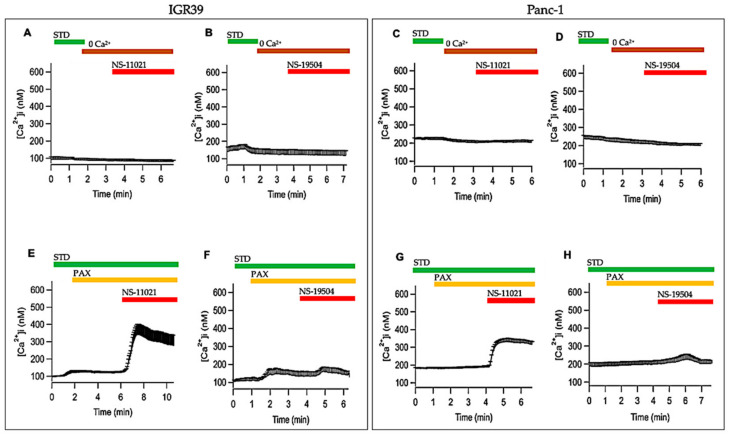
Effects of calcium free bath solution or paxilline on NS-11021- and NS-19504-induced calcium increase. Average [Ca^2+^]_i_ signal in IGR39 (**A**,**B**) and Panc-1 (**C**,**D**) cells after the application of 10 µM NS-11021 or NS-19504 in STD 0 Ca^2+^. Average [Ca^2+^]_i_ signal in IGR39 (**E**,**F**) and Panc-1 (**G**,**H**) cells after the application of 10 µM NS-11021 or NS-19504 in STD solution containing 1 µM PAX. Data ± SE are 220 ± 55 nM (*n* = 38) for IGR39 and 145 ± 20 nM (*n* = 45) for Panc-1. Horizontal bars indicate the time period of exposure. (*n*) refers to the number of cells.

Since BK channel gating is strongly affected by intracellular calcium levels, we investigated whether activation of BK channel in IGR39 and Panc-1 cells was directly or indirectly mediated by an increase of intracellular calcium levels using FURA-2 Ca^2+^ imaging. Surprisingly, application of NS-11021 induced a dramatic increase of intracellular calcium levels in IGR39, as well as in Panc-1 cells (Figure 8A,D). In parallel, although NS-11021 failed to activate BK current in IGR37 (Figure S2), it was still able to induce a small calcium increase in this cell line (Figure 9G). Instead, NS-19504 perfusion only produced a moderate increase of intracellular calcium levels in both IGR39 and Panc-1 (Figure 8B,E). Conversely, no calcium increase was observed when IGR37 cells were perfused with 20 µM of NS-19504 (Figure 8H).

To clarify whether intracellular calcium increase was due to an influx of extracellular calcium, we performed experiments using a bath solution without Ca^2+^ and with 3 mM EGTA (STD 0 Ca^2+^). In these conditions, Ca^2+^ increase induced by BK activators was completely absent in both IGR39 and Panc-1 cells (Figure 9A–D), demonstrating that Ca^2+^ influx underlies the observed calcium increase. Activation of BK channels presumably leads to membrane hyperpolarization, and consequently to an increase of the driving force for calcium influx across the plasma membrane. To investigate whether the intracellular calcium increase was dependent on hyperpolarization, we repeated the experiments in the presence of a specific BK channel inhibitor, i.e., paxilline [32]. Figure 9E,G shows that paxilline only reduced the intracellular calcium increase by 30% and 60%, induced by NS-11021 in IGR39 and Panc-1 cells (see Figure 8A,B). These data suggest a direct, BK channel-independent effect of the compound on calcium entry pathways in both cell lines. Vice versa, paxilline almost completely blocked calcium increase induced by NS-19504 in both cell lines (Figure 9F,H). Thus, these findings indicate that for NS-19504, the calcium entrance is mostly due to BK channel-mediated hyperpolarization, while other membrane conductance is involved in calcium increase caused by NS-11021.

**Figure 10:**
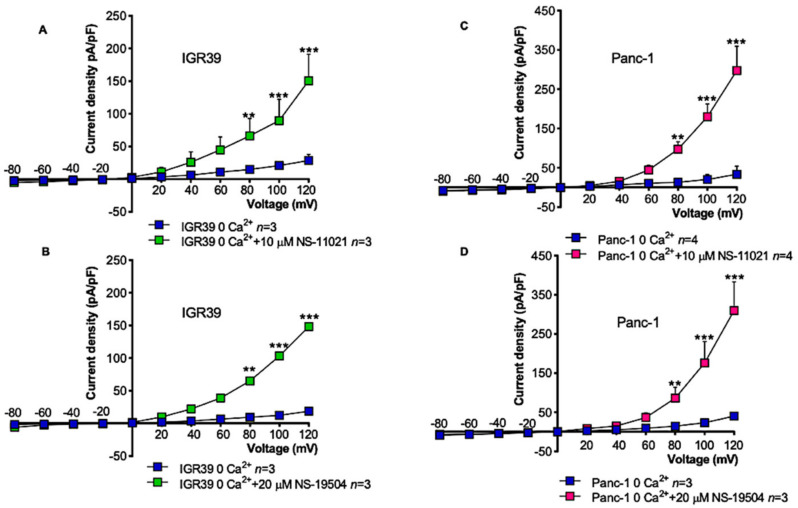
Effects of BK channel openers in absence of extracellular calcium in IGR39 and Panc-1 cells. Current density-to-voltage relationship measured in whole-cell patch-clamp experiments. The voltage protocol consisted of voltage steps from −80 to +120 mV in +20 mV increments from a holding potential of −50 mV. The duration of the voltage steps was 500 ms. Exposure to 10 µM NS-11021 (**A**,**C**), or alternatively 20 µM NS-19504 (**B**,**D**) induced BK current activation in both IGR39 and Panc-1 cells. ** *p* < 0.01 and *** *p* < 0.001, paired Student’s *t*-test. (*n*) refers to the number of cells.

To further test the role of Ca^2+^ influx as trigger of BK channel activation, we measured currents in response to the activators in the absence of extracellular Ca^2+^. Importantly, NS-11021 and NS-19504 promoted the activation of BK channel in both cell lines (Figure 10), demonstrating that both compounds are able to directly activate BK.

## 4. Discussion

In the present study, we employed different BK channel activators [20,22,33,34] (Figure 1) in order to explore the feasibility to modulate BK channel function to reduce cell viability, proliferation, and migration in two different cancer lines, i.e., melanoma (IGR39) and PDAC (Panc-1). As a negative control, to test whether effects were specific to BK channel activation, we utilized the IGR37 cells, as in this cell line, BK is barely expressed compared to its primary counterpart IGR39 [30].

Here, we confirmed that 10 µM NS-11021 increases BK outward currents in both IGR39 and Panc-1 cell lines (Figure 2 and Figure 3), but not in IGR37 cells (Figure S2). These results are consistent with a study on effects of NS-11021 on BK heterologously expressed in *Xenopus laevis* oocytes and in HEK-293 cells [33]. As reported by Rockman et al. [35], NS-11021-induced activation reflects an increase in channel open probability independent of Ca^2+^ binding. This latter aspect was further demonstrated by using a truncated BK channel. In this form, the CSD subunit was removed and replaced with a short amino acid sequence so that the channel could be activated by voltage but no longer by Ca^2+^ due to the lack of CSD [36]. In parallel, we found that NS-19504 also favors the opening of BK channels in IGR39 and Panc-1 cells (Figure 2). Instead, a third BK channel opener, BMS-191011 (Figure 1) [22], showed only very poor potency: application of 20 or 40 µM BMS-191011 activated BK channels in less than 50% IGR39 cells (Figure 4) and led to less than twofold potentiation in Panc-1 cells (Figure 4). In addition, in IGR39 cells, BMS-191011 promoted the activation of a voltage-independent K^+^ conductance at negative voltages (Figure 4B), not responsive to paxilline. Because of the low potency and the ambiguous properties of BMS-191011, we did not test this compound in cell viability, proliferation, and migration assays. The very small effect of BMS-191011 on BK is in contrast with previous results [13,21,23]. 

Encouraged by the potent effects of NS-11021 and NS-19504 on BK channel activity in IGR39 and Panc-1 cells, we explored whether the compounds were able to reduce cell viability, migration, and proliferation. Rather surprisingly, only NS-11021 had significant effects on these parameters in both IGR39 and Panc-1 cells (Figure 5, Figure 6 and Figure 7). To better clarify whether NS-11021-induced cell death or proliferation/migration inhibition specifically acted via opening of BK channels, we used the metastatic melanoma IGR37 cell line that effectively lacks BK channel activity as a negative control (Figure S2) [30]. Surprisingly, NS-11021 induced both cell death and proliferation inhibition in this cell line (Figure 5 and Figure 7). This result suggests that the effects of NS-11021 are not only specifically dependent of opening of BK channels. We discovered a possible mechanism underlying BK-independent effects of NS-11021 by assaying the intracellular Ca^2+^ concentration. Indeed, both NS-11021 and NS-19504 led to an increase of [Ca^2+^]_i_ in IGR39 and Panc-1 cells that was strictly dependent on extracellular calcium (Figure 8). However, while NS-19504-induced calcium influx was abolished by BK channel inhibitors (paxilline (Figure 9) and tetraethylammonium), NS-11021-induced calcium influx was mostly retained in the presence of BK blockers, strongly suggesting that NS-11021 activates a calcium-permeable membrane conductance independent of BK. Consistent with this interpretation, we found that NS-11021-mediated current activation was dramatically reduced when experiments were carried out in the absence of extracellular calcium (Figure 10). Altogether, the observations show that NS-11021 has a double effect: a direct activation of BK channels, and an increase of intracellular calcium, probably via the activation of a calcium permeable conductance. The calcium increase further activates BK currents beyond the direct effect. In IGR37 cells, the observed NS-11021-induced calcium increase is BK independent, as these cells express the BK encoding gene at very low levels [30] and do not show BK channel activity, even in the presence of NS-11021 (Figure S2). In contrast, we did not detect an unspecific effect of NS-19504 on intracellular calcium levels. Thus, among the activators tested in our work, NS-19504 is the only one that potently activates BK channels, in agreement with other authors [20,37,38], possibly without further off-target effects. Importantly, this specific activator had no significant effects on cell viability, migration, or proliferation in any of the three cell lines tested.

Consequently, we hypothesized that the effects of NS-11021 of viability, proliferation, and migration might be mostly ascribed to the increase of intracellular calcium levels observed in our cells and the multitude of downstream pathways it can activate. These data are in line with what reported by other authors, namely, that some substances (phloretin, NS-004, or NS-1619) that are able to open BK channels could also cause an increase of intracellular Ca^2+^ levels [39,40]. To this end, a recent study, performed by Zuccolini and co-authors, proposed a similar mechanism for DCPIB, a well-known blocker of volume-regulated anion channels (VRACs) [41]. According to the authors, DCPIB favors the activation of BK channel, in parallel bringing about a rapid intracellular calcium influx in both IGR39 and Panc-1 cells [31].

Many reports have pointed out that the activation of BK channels has a relevant role in reducing viability, proliferation, and migration of cancer cells. For example, a recent study investigated the role of BK in the regulation of the apoptosis in ovarian cancer cells by employing NS-1619; the latter was able to induce apoptosis in A2780 cells [42]. These results indicate that the activation of BK channels by NS-1619 play an important role in regulating proliferation of human ovarian cancer cells, and could induce apoptosis through increase of protein expression levels of p53, p21, and Bax. Unfortunately, no evidence has been reported about calcium signals in order to confirm the specific anticancer activity of BK channel activation by NS-1619. In a later study, it has been reported that activation of BK channels by NS-11021 or BMS-191011 induced both cell cycle arrest in G2 phase and migration, as well as apoptosis via caspase-3 activation in breast cancer cells [13]. Moreover, in a triple-negative breast cancer (TNBC)-induced xenograft model, treatment with BMS-191011 significantly slowed cancer growth [13]. These data appeared to support the idea that hyperpolarization induced by BK channel openers could be a novel pharmacological approach in TNBC patients. However, our results on NS-11021 and BMS-191011 shed considerable doubt on the conclusion of a causal involvement of BK channels in these studies. Similar effects were also obtained in glioblastoma cells, where BK channel activation by phloretin and NS-1619 reduced the migration velocity by about 50% [39]. However, unspecific effects have not been completely ruled out in that study either.

In contrast, it has been demonstrated that specific inhibitors of BK channel could suppress cancerous behaviors, although this does not seem to apply to all studies. In particular, block of BK by iberiotoxin or RNAi significantly inhibited cell proliferation of PC-3 cells [43]. Moreover, knocking down KCNMA1 reduced proliferation by 58% in T47D breast cancer. Similarly, pharmacological inhibition of BK channels by paxilline decreased cell proliferation rate by 54% [38]. In a later investigation, upregulation of KCNMA1 was associated with greater invasiveness and trans-endothelial migration, both of which could be attenuated by blocking the channel [15]. Along these lines, Rosa and collaborators have shown that chronic hypoxia functionally upregulates BK channels in glioblastoma cells, and this upregulation contributes to at least two aspects of the aggressive phenotype that these cells acquire under hypoxic conditions, namely, cell migration and chemo-resistance. However, these effects could be prevented by BK channel inhibition [44]. Thus, although channel activity of BK can be effectively modulated by activators and blockers, these contradictory findings support the idea that BK channel might function in cancer biology depending on the cell model used [45,46] and on modulating calcium influx.

## 5. Conclusions

In the present study, we clearly demonstrated that both cancer cell lines (IGR39 and Panc-1) exhibit increased BK channel activity following treatment with BK activators (NS-11021/NS-19504), while BMS-191011 was almost ineffective. In addition, we report that NS-11021 leads to a BK-independent increase of intracellular Ca^2+^ concentration, thus complicating any interpretation of its role as specific BK channel activator. 

Indeed, we strongly discourage the use of the presumably specific BK channel openers NS-11021 and BMS-191011 for investigation of the potential of BK channel modulation as a means to reduce cancer progression. 

Employing the more specific activator NS-19504, our study revealed for the first time that *KCNMA1* does not directly exhibit an oncogenic potential for analyzed cell lines. However, since the role of BK channels in human cancer is a very complex one and may not be a universal one, further studies on the function of BK channels in pathophysiological processes, such as Ca^2+^ entry, are needed to unmask the molecular mechanism by which BK channel could modulate events related to cancer. 

In conclusion, our results raise an important alarm flag regarding the use of potential specific BK channel openers as anti-cancer agents. However, the possibility of determining the molecular or biophysical features of ionic channels can help in designing outstanding therapeutic strategies that combine the easy accessibilities of ion channel molecule with their modulation, and, hopefully, with the absence of potentially harmful side effects. 

## Figures and Tables

**Table 1:** Composition of extracellular solutions (pH 7.3, adjusted with NaOH, and 300 mOsm/kg H_2_O).

Variable	NaCl	KCl	CaCl_2_	MgCl_2_	HEPES	EGTA
STD	145 mM	5 mM	2 mM	1 mM	10 mM	-
STD 0 Ca^2+^	145 mM	5 mM	-	1 mM	10 mM	3 mM

## Data Availability

The data that support the findings of this study are available from the corresponding author upon reasonable request.

## Supplementary Materials

The following are available online at https://www.mdpi.com/article/10.3390/cancers13236144/s1, Figure S1: Reversible effect of NS-19504 on currents in IGR39 and Panc-1 cells, Figure S2: Effect of NS-11021 on membrane current in IGR37 cells.
