# Supplementary material for: NS-11021 Modulates Cancer-Associated Processes Independently of BK Channels in Melanoma and Pancreatic Duct Adenocarcinoma Cell Lines
Source: Cancers (Basel). 2021 Dec 6;13(23):6144. doi: 10.3390/cancers13236144 (PMC8656804; doi:10.3390/cancers13236144)
Supplement: Supplementary file 1 [file cancers-13-06144-s001.zip › cancers-1459183-supplementary.pdf]

Supplementary Materials: NS-11021 Modulates Cancer-Associated Processes Independently of BK Channels in Melanoma and Pancreatic Duct Adenocarcinoma Cell Lines

Alessia Remigante, Paolo Zuccolini, Raffaella Barbieri, Loretta Ferrera, Rossana Morabito, Paola Gavazzo, Michael Pusch and Cristiana Picco

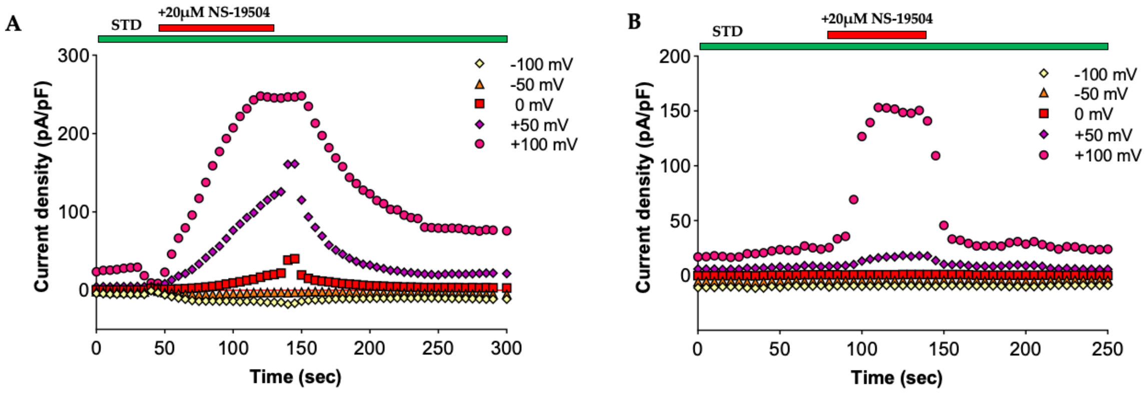

Figure S1. Reversible effect of NS-19504 on currents in IGR39 and Panc-1 cells. Exemplary plots of current amplitudes measured every 5 seconds at different voltages (−100 mV, −50 mV, 0 mV, +50 mV, +100 mV) in representative IGR39 (**A**) and Panc-1 (**B**), respectively. Green indicates STD solution, while red indicates STD solution + 20 µM NS-19504.

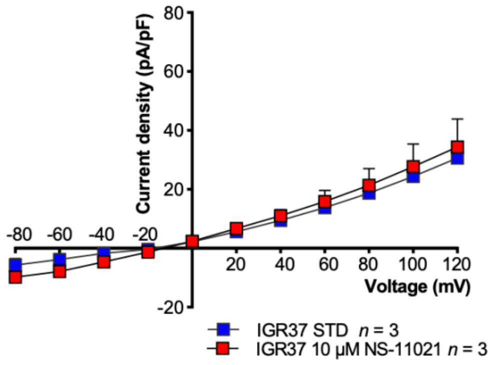

Figure S2. Effect of NS-11021 on membrane current in IGR37 cells. Current density-voltage relationship measured in whole-cell patch-clamp experiments before and after NS-11021 application. Ns versus STD condition, as determined by paired Student's *t*-test. (*n*) refers to the number of cells.
